# Epidemiological aspects and clinical outcome of patients with Rhinocerebral zygomycosis: a survey in a referral hospital in Iran

**DOI:** 10.11604/pamj.2016.24.232.9688

**Published:** 2016-07-13

**Authors:** Vida Bozorgi, Mahshid Talebitaher, Neda Shalbaf, Nima Radmanesh, Fatemeh Nasri, Mohammad Mostafa Ansari-Ramandi

**Affiliations:** 1 Infectious disease department, Rasoul-e-Akram Hospital, Iran University of Medical Sciences, Tehran, Iran; 2 Rajaie Cardiovascular Medical and Research Center, Iran University of Medical Sciences, Tehran, Iran

**Keywords:** Rhinocerebral zygomycosis, in-hospital mortality, morbidity

## Abstract

**Introduction:**

No comprehensive reports have been published on epidemiological status of Rhinocerebral zygomycosis infections and its outcome in our population, Hence, the current study came to address epidemiological characteristics as well as clinical outcome of patients with Rhinocerebral zygomycosis infection referred to a referral hospital in Iran.

**Methods:**

This retrospective study was performed at the Rasoul-e-Akram hospital, an 800-bed tertiary care teaching hospital in Tehran, Iran. The pathology recorded charts were reviewed to identify all cases of Rhinocerebral zygomycosis from patients admitted between April 2007 and March 2014. A diagnosis of Rhinocerebral zygomycosis was based on histopathological assessments.

**Results:**

Sixty four patients with Rhinocerebral zygomycosis were assessed. The mean age of the patients was 46.07 ± 22.59 years and 51.6% were female. Among those, 67.2% were diabetic, 26.6% were hypertensive and 29.7% had history of cancer. Different sinuses were infected in 73.4% of the patients. Out of all the patients 26.6% underwent surgical procedures and 17.2% were controlled medically. Extensive debridement was carried out in 40.6%. Neutropenia (<1500 cell/ µl) was revealed in 12.5%. In-hospital mortality rate was 35.9% and prolonged hospital stay (> 14 days) was found in 60.9%. According to the Multivariable logistic regression analysis, the main predictors of in-hospital mortality included female gender, advanced age, the presence of sinus infection, and neutropenia, while higher dosages of amphotericin administered had a protective role in preventing early mortality. In a similar Multivariate model, history of cancer could predict prolonged hospital stay, whereas using higher dose of amphotericin could lead to shortening length of hospital stay.

**Conclusion:**

There is no difference in demographic characteristics between our patients with Rhinocerebral zygomycosis and other nations. The presence of diabetes mellitus is closely associated with the presence of this infection. Sinus involvement is very common in those with Rhinocerebral zygomycosis leading to high mortality and morbidity. Besides female gender, advanced age, and presence of neutropenia was a major risk factor for increasing early mortality. The use of higher doses of antifungal treatment such as amphotericin can prevent both mortality and prolonged hospital stay. The cancer patients may need longer hospital stay because of needing comprehensive in-hospital treatment.

## Introduction

Zygomycosis refers to infectious disease variants caused by Mucorales fungi species [1]. It may occur in progressive underlying risk profile patients such as immunosuppression, diabetic ketoacidosis, malignancies, extreme malnutrition, any conditions led to iron overload, burns, and trauma. Zygomycosis has a life-threatening nature with multi-organ involvements such as cutaneous, pulmonary, and even neurologic invasions [2]. Thus, proper management and treatment of this multi-infection needs early detection of underlying risk factors along with medical and even surgical aggressive therapies. However, misdiagnosing identifiable risk factors is now a major problematic issue to improve disease progression and outcome [3, 4]. Moreover, because of identified different routes of infection such as inhalation of conidia, gastrointestinal ingestion (among malnourished patients), non-sterile tape and contaminated wooden splints, traumatic inoculation, and even via natural disasters [5–9], the problem is compounded for disease control and treatment. Descriptions of zygomycosis appear to be increasing, perhaps given higher numbers of persons at risk [10]. Epidemiologically, the infection by zygomycosis has a worldwide distribution with no known gender, age, or interracial preferences; however some institutional studies have shown a gender tendency with a male-to-female ratio of 3:1 [11]. Regarding disease prognosis, the presence of some serious comorbidities and needing aggressive coordinated treatment strategies may potentially affect prognosis, however despite all efforts to identify disease risk factors as well as to control disease progress, zygomycosis may carry a mortality rate of 50-85% mostly due to its serious consequences including rhinocerebral and pulmonary events [12–14]. Unfortunately, no comprehensive reports have been published on epidemiological status of mucormycosis infections and its outcome in our population from 2007, Hence, the current study came to address epidemiological characteristics as well as clinical outcome of patients with mucormycosis infection referred to a referral hospital in Iran.

## Methods

This retrospective study was performed at the Rasoul-e-Akram hospital, an 800-bed tertiary care teaching hospital in Tehran, Iran. The pathology recorded charts were reviewed to identify all cases of zygomycosis from patients admitted between April 2007 and March 2014. Patients were included in the study if they met the criteria for proven invasive Rhinocerebral zygomycosis based on the revised definitions of invasive fungal disease of the European Organization for Research and Treatment of Cancer/Mycosis Study Group (EORTC/MSG) [15]. In total, 91 patients were identified that among them, 16 were excluded because of discharge against medical advice. The cases that were diagnosed on an outpatient basis or on the day of surgery and were not admitted to either hospital were also excluded. Data was collected on patients´ demographics, underlying conditions, concomitant immunosuppressive medications, laboratory data, radiologic findings, clinical features, antifungal treatment, surgical procedures, and outcomes. A diagnosis of Rhinocerebral zygomycosis was based on histopathological demonstration of broad, ribbon-like, wide-angled branching, non-septate hyphae even in the absence of positive cultures, and accompanying tissue invasion by fungal hyphae [16, 17]. The zygomycosis genus was determined by morphological examination of conidia, hyphae, and whole colonies. The study endpoint was first to assess in-hospital mortality and length of hospital stay and second was to determine main predictors of mortality and prolonged hospital stay in the patients. Results were presented as mean ± standard deviation (SD) for quantitative variables and were summarized by frequency (percentage) for categorical variables. Continuous variables were compared using t test or Mann-Whitney U test whenever the data did not appear to have normal distribution or when the assumption of equal variances was violated across the study groups. Categorical variables were, on the other hand, compared using chi-square test. The multivariable regression models were applied to determine indicators of disease outcome. For the statistical analysis, the statistical software SPSS version 16.0 for windows (SPSS Inc., Chicago, IL) was used. P values of 0.05 or less were considered statistically significant.

## Results

**Figure 1:**
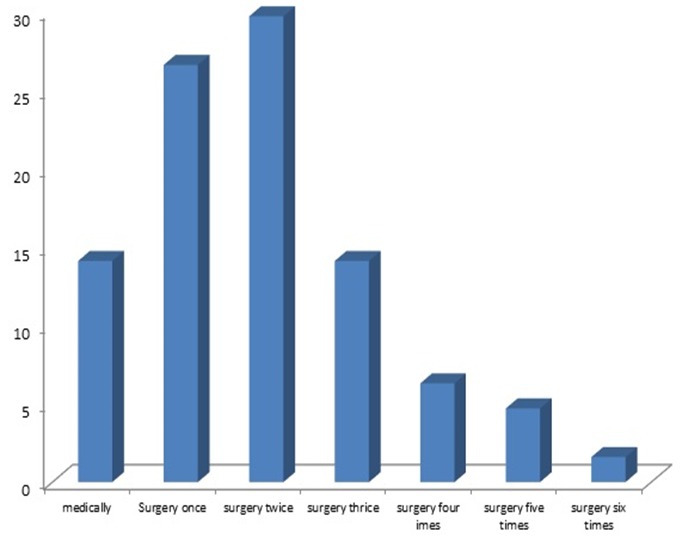
Treatment approach in patients with sinus involvement related to mucormycosis

In total, 64 patients with Rhinocerebral zygomycosis were assessed. The mean age of the patients was 46.07 ± 22.59 years (ranged 4 to 87 years) from which 51.6% were female. The mean age of affected men was similar to diseased women (45.89 ± 26.36 years versus 46.24 ± 18.80 years, p = 0.950). The overall prevalence of malignant conditions was 29.7%, 67.2% were diabetic, and 26.6% were hypertensive. The average dose of amphotericin in administered patients was 2135.64 ± 1870.91 mg. Different sinuses were infected in 73.4%, 26.6% underwent surgical procedures once and 56.2% underwent these procedures more than once, and 17.2% were controlled medically (Figure 1). Functional endoscopic sinus surgery (FESS) was also done on 53.1% of patients. Eye enucleating was considered for 18.8% of cases. Regarding diagnostic approaches, pathological assessment was planned for 85.9% and 25.0% were also assessed by endoscopy. Extensive debridement was carried out in 40.6% as a surgical procedure. The mean WBC count of the patients was 7947.60 ± 5446.86 cells per microliter (µl) of blood that neutropenia (<1500 cell/ µl) was revealed in 12.5%. In total, in-hospital mortality rate was 35.9%. The mean length of hospital stay was 26.94 ± 24.70 days that prolonged hospital stay (> 14 days) was found in 60.9% of the patients. According to the Multivariable logistic regression analysis (Table 1), the main predictors of in-hospital mortality included female gender (OR = 5.263, P = 0.002), advanced age (OR = 1.063, P = 0.001), the presence of sinus infection (OR = 4.836, P = 0.018), and neutropenia (OR = 31.250, P = 0.003), while higher dosages of amphotericin deoxycholate administered had a protective role in preventing early mortality (OR = 0.825, P = 0.025). In a similar Multivariate model (Table 2), history of cancer could predict prolonged hospital stay (OR = 8.413, P = 0.043), whereas using higher dose of amphotericin could lead to shortening length of hospital stay (OR = 0.998, P < 0.001).

**Table 1:** Main predictors of in-hospital mortality

Variable	B	S.E.	Wald	p-value	OR	95.0% CI for OR	
						Lower	Upper
Female gender	1.662	.544	9.338	0.002	5.263	1.815	15.385
Age	0.060	.018	11.099	0.001	1.063	1.026	1.101
Diabetes	-0.083	.980	0.007	0.932	0.920	0.135	6.283
Hypertension	-0.107	.484	0.049	0.825	0.898	0.348	2.319
Amphotericin use	0.226	0.568	5.021	0.025	0.825	0.295	0.979
Sinus infection	1.576	1.665	5.610	0.018	4.836	1.312	17.822
Neutropenia	-3.451	1.157	8.898	0.003	31.250	3.268	333.33
Cancer	0.036	0.662	0.003	0.956	1.037	0.284	3.794
Constant	7.912	2.392	10.944	0.001	2.730		

**Table 2:** Main predictors of prolonged hospital stay (LOS > 14 days)

Variable	B	S.E.	Wald	p-value	OR	95.0% CI for OR	
						Lower	Upper
Female gender	-1.116	0.656	2.888	.089	0.328	0.091	1.186
Age	0.034	0.020	2.846	.092	1.035	0.995	1.076
Diabetes	0.324	1.138	0.081	.776	1.383	0.149	12.880
Hypertension	0.778	0.734	1.123	.289	2.178	0.516	9.184
Amphotericin use	-0.002	0.000	22.696	.000	0.998	0.998	0.999
Sinus infection	1.239	0.800	2.399	.121	3.453	0.720	16.571
Neutropenia	0.258	1.056	0.060	.807	1.294	0.163	10.250
Cancer	2.130	1.052	4.096	.043	8.413	1.070	66.169
Constant	-1.782	2.418	0.543	.461	0.168		

## Discussion

As the first results and regarding demographic characteristics of our patients who suffered Rhinocerebral zygomycosis, we showed the prevalence of disease in an age wide spectrum from childhood to old adulthood. Also, men and women were similarly affected by this infection. Among underlying risk profiles, high prevalence of diabetes mellitus was revealed among patients that was shown in about two-third of them. Epidemiological studies showed high prevalence of diabetes mellitus in the patients in both developed and developing nations, however some clear differences were also revealed in the epidemiological aspects of disease between the nations that in developed countries, the disease remains uncommon and mostly occur in patients with diabetes mellitus and hematological malignancies, while in developing countries, zygormycosis has a sporadic pattern closely related to uncontrolled diabetes or trauma [18–20]. It seems that the relationship between high incidence rate of zygomycosis and diabetes is mainly influenced by uncontrolled situation of diabetes or occurring ketoacidosis as a serious predisposing factor for this infection. In total, reviewing the literature demonstrated the risky role of diabetes mellitus in 36% to 88% of patients [20–25]. In contrast, controlling diabetes, proper management of ketoacidosis and the use of statins as a main treatment protocols for metabolic syndrome could effectively reduce the incidence of zygomycosis [26–28]. Sinuses involvement is a common clinical manifestations affecting disease poor prognosis. This complication commonly appears as rhinocerebral mucormycosis, that is manifested by either sinusitis or periorbital cellulitis [29]. If untreated, sinusitis can spread from the sinuses and extend into the neighboring tissues such as orbit or orbital muscular complex leading to potential visual complications or may extend into the mouth and produce painful, necrotic ulcerations of the hard palate [30]. Another serious complication can be the spreading infection from sinus posteriorly to central nervous system and leading to cerebral vascular invasion and cerebral infarction, a serious cause for increasing disease-related mortality [31]. In our observation, 73.4% of patients suffered sinuses infections that mostly underwent invasive surgical procedure to prevent its progression. More importantly, the presence of sinusitis has been identified as an important predicting factor for early mortality in these patients leading to4.8 times more risk for mortality.

We saw an early mortality of 35% for affected patients with Rhinocerebral zygomycosis that seems to be considerably high despite performing curative management in our center. Naturally, the early and long-term mortality following Rhinocerebral zygomycosis is strongly associated with the presence of underlying comorbidities. It has been shown that the overall mortality of pulmonary mucormycosis is approximately 50 to 70% [32, 33]. Cutaneous and subcutaneous disease may lead to necrotizing fasciitis, which has a mortality approaching 80% [34, 35]. The mortality associated with dissemination to the brain approaches 100% [36]. In this regard, managing disease by proper antifungal agents leads to considerably decrease in mortality. It has been indicated that in cases with rhinocerebral mucormycosis, the mortality is about 70% in cases treated with antifungal agents alone versus 14% in cases treated with antifungal agents plus surgery [37, 38]. Similarly, in a combined series of rhinocerebral, cutaneous, and pulmonary mucormycosis, 65% of patients treated with surgery plus antifungal agents survived the infection, compared to none of the patients treated with antifungal agents alone [39]. Thus, the death rate is directly associated with whether the patients were managed medically or surgically. In our study, regardless of the type of comorbidity or treatment approach, high early mortality was reported following Rhinocerebral zygomycosis infection. However, as shown in our multivariable analysis, administrating antifungal treatment by high dose amphotericin, not only reduced in-hospital mortality, but also shortened hospital stay. However, it seems that the rate obtained for early mortality is notably high requiring deep assessment of its causes among our population.

Two underlying conditions including history of cancer and presence of neutropenia can adversely affect outcome of Rhinocerebral zygomycosis as shown in our analysis. It has been clearly shown that the overall mortality rate for Rhinocerebral zygomycosis remains higher than 50%, and it approaches 100% among patients with persistent neutropenia [32, 40]. In fact, the neutrophils are critical for inhibiting fungal spore proliferation and thus decrease in neutrophil count can predispose the patients to progressive Rhinocerebral zygomycosis. The risky role of neutropenia is more prominent in patients with malignancies especially hematological cancers with immunosuppressive patterns.

## Conclusion

In summary, comparing our results with data previously reported in the literature shows similarities in demographic distribution of Rhinocerebral zygomycosis in our population. Among common comorbidities, the presence of diabetes mellitus is closely associated with the presence of this infection because more than two-third of affected patients with this infection are diabetics. Sinus involvement is very common in those with Rhinocerebral zygomycosis even in early stages of disease leading to high mortality and morbidity in the patients needing invasive surgical treatments. Besides female gender, advanced age, and presence of neutropenia (an indicator for immunosuppression) act as a major risk factor for increasing early mortality, the use of antifungal treatment such as high dose amphotericin can prevent both mortality and prolonged hospital stay. The cancer patients may need longer hospital stay because of needing comprehensive in-hospital treatments.

### What is known about this topic

- Presence of diabetes mellitus is closely associated with rhinocerebral zygomycosis;
- Neutropenia is a risk factor for early mortality in patients with rhinocerebral zygomycosis

### What this study adds

- High dose amphotericin can reduce both mortality and prolonged hospital stay in patients with rhinocerebral zygomycosis;
- Cancer patients need longer hospital stay for treatment of rhinocerebral zygomycosis
